# Description and validation of a portable system for biomechanical ex vivo knee kinematics and laxity assessment in simulated intra‐operative scenarios

**DOI:** 10.1002/jeo2.70756

**Published:** 2026-05-25

**Authors:** Mattia Sisella, Lorenzo Maggi, Benedetto Allotta, Bernardo Innocenti

**Affiliations:** ^1^ BEAMS Department (Bio Electro and Mechanical Systems), École Polytechnique de Bruxelles Université Libre de Bruxelles Bruxelles Belgium; ^2^ Department of Industrial Engineering University of Florence Florence Italy

**Keywords:** ex vivo testing, knee biomechanics, ligaments laxity, patello‐femoral kinematics, tibio‐femoral kinematics

## Abstract

**Purpose:**

The analysis of knee joint biomechanics under intraoperative‐like conditions is fundamental to support surgical decisions and improve clinical outcomes. Many experimental systems developed in recent years are characterized by high complexity and limited portability, which may restrict the reproduction of surgical intraoperative workflows. Therefore, this study describes the design and experimentally validates a compact and portable testing platform capable of accurately analysing knee tibio‐femoral and patello‐femoral kinematics and laxity under intraoperative‐like conditions.

**Methods:**

The system integrates motion capture, mechanical fixation, motor‐driven actuation, force measurement and dedicated software into a unique platform. It enables passive and active open‐chain knee motions, as well as standardized ligament laxity testing. Tibio‐femoral and patello‐femoral kinematics are simultaneously recorded while quadriceps and external forces are measured respectively through mono‐axial and tri‐axial load cells. System accuracy and repeatability were assessed through load cell calibration and dynamic testing on a synthetic knee model. Transportability, setup time and overall performance were evaluated under realistic ex vivo experimental conditions.

**Results:**

Validation demonstrated close agreement between set and measured loads, with measurement accuracies of approximately 1% for quadriceps forces and 0.75% for externally applied knee forces. Motor‐driven motion showed high repeatability, with mean standard deviations of 0.51° for flexion–extension cycles and 1.62° for multiple dynamic flexion tests. Motion capture calibration achieved a pointer tip positional error of approximately 0.20 mm. Ex vivo testing confirmed reliable reproduction of knee motion and highly reproducible measurement, with rapid set‐up and workflow smoothness.

**Conclusion:**

The system represents a portable, accurate, repeatable and validated experimental platform for biomechanical assessment of knee joint kinematics and laxity under intraoperative‐like conditions. Its modular design, rapid setup and capability to combine kinematic and kinetic measurements make it a valuable tool for investigating the biomechanical impact of surgical techniques or implants, and patient‐specific joint behaviour in ex vivo settings.

**Level of Evidence:**

N/A.

**Abbreviations**
AP: antero‐posterior
DDF: double dynamic flexion
FE: flexion–extension
FS: full scale
IE: internal–external
ML: medio‐lateral
TKA: total knee arthroplasty
VV: varus–valgus

## INTRODUCTION

The analysis of knee biomechanics plays a crucial role in the clinical comprehension of knee joint function and performance. The complex biomechanics of the knee arise from the three‐dimensional interaction between the tibio‐femoral and the patello‐femoral joints [16], and are strongly dependent on the anatomy and the soft tissue condition of the specific subject [1, 16]. The biomechanical complexity becomes particularly relevant in the context of total knee arthroplasty (TKA), where the replacement of the articular surfaces and the alteration of ligamentous constraints may substantially modify physiological joint kinematics, possibly leading to different motion patterns and clinical outcomes. Experimental testing on ex vivo specimens has proven to be a reliable method to evaluate the kinematical behaviour of both native and prothesized knees under controlled conditions [1, 3, 18]. In recent years, several devices have been developed to evaluate knee biomechanics in both closed‐chain and open‐chain configurations [3]. Since the creation of the renowned Oxford test rig [24], numerous mechanical test rigs have been introduced [14, 19, 20, 21, 22], while the advancements in robotics over the last few years have further facilitated the development of devices based on robotic manipulators. These devices have been used for both ex vivo knee testing [6, 11, 23] and in vivo, non‐invasive knee laxity measurements [17]. They allow a very accurate (<0.2 mm and 0.2°) application of displacement and rotation [10, 12, 23], making possible the analysis of the knee joint behaviour in different conditions as well as the evaluation of orthopaedic implants design, as highlighted in several studies that have been conducted in the last years [4, 7, 9]. However, such experimental systems often require complex control procedures and extensive calibration, which limit their accessibility and reduce the reproducibility of results across laboratories. In addition, these devices are typically highly sophisticated and bulky, making them difficult to transport. This restricts their broad applicability for knee kinematics testing in different settings, such as cadaveric laboratories, research facilities of orthopaedic implant manufacturers or environments already occupied by other large equipment, for example, surgical robots. Furthermore, the surgeon plays a central role in determining the clinical outcome of a TKA; therefore, surgical decisions should be supported by a direct understanding of how individual choices influence knee kinematics and overall joint behaviour, ideally through systems that seamlessly integrate surgical practice with experimental testing. However, such portable, surgeon‐oriented experimental systems are still lacking. Therefore, this study presents the design of a new fully portable automated testing platform for ex vivo knee specimens, capable of analysing knee kinematics under intraoperative‐like conditions. The primary objective is to validate the spatial and force measurement accuracy of the system, together with its repeatability, practical applicability and ease of use under realistic experimental workflows.

## MATERIALS AND METHODS

### System overview

**Figure 1:**
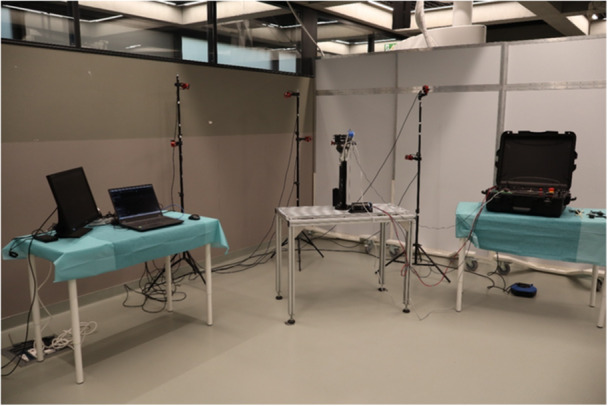
Overall experimental set‐up with four modules.

The system (Figure 1) has been designed as a modular platform composed of 4 semi‐independent functional modules, which can be used either individually or in combination, depending on the type of experimental tests to be performed. The overall experimental workflow integrates motion capture, mechanical actuation, force measurement and software control into a single portable setup, allowing seamless transition between testing and surgical phases.

### Motion capture module

**Figure 2:**
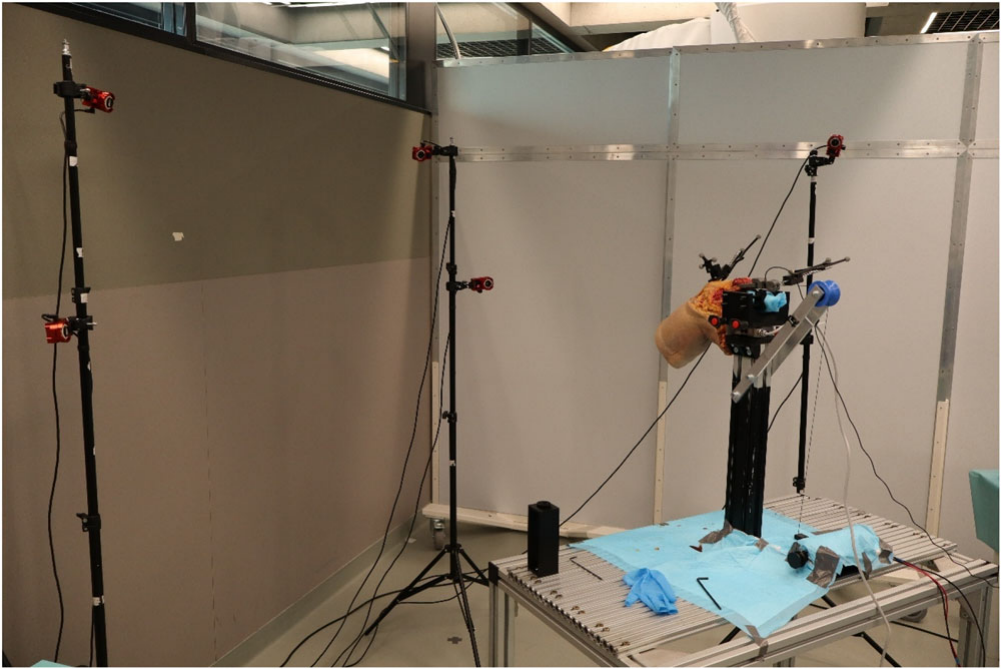
Motion capture module.

The motion capture module (Figure 2) is based on six OptiTrack Flex 13 cameras (NaturalPoint Inc.) and the associated Motive software (NaturalPoint Inc.). The cameras are mounted on tripods and arranged in a semicircular configuration around the working volume containing the specimen, with the layout adaptable to the available space. To maximize capture volume and reduce marker occlusion, the cameras are distributed across two vertical levels: three cameras are positioned at the level of the specimen, while the remaining three are placed approximately 600 mm above. Prior to each testing session, a full camera calibration is performed, and the setup is accepted only when the overall final error is below or equal to 0.2 mm. Infrared reflective markers are mounted on custom‐designed marker‐sets to enable tracking of the three bones of the knee joint. The marker sets are positioned on the medial side and arranged in a planar configuration to ensure optimal visibility toward the cameras. Femoral and tibial marker sets are rigidly fixed to the corresponding bone using, respectively, two 3.2 mm threaded surgical pins inserted bi‐cortically to ensure stable fixation, while patella fixation is achieved using two self‐tapping screws. Each marker set incorporates a fast‐release mechanism, allowing the marker assembly to be temporarily removed while preserving the bone‐mounted reference, thereby enabling surgical procedures to be performed without loss of kinematic reference. A custom‐made digitizing probe was developed to acquire anatomical landmark positions during the registration phase.

### Mechanical module

**Figure 3:**
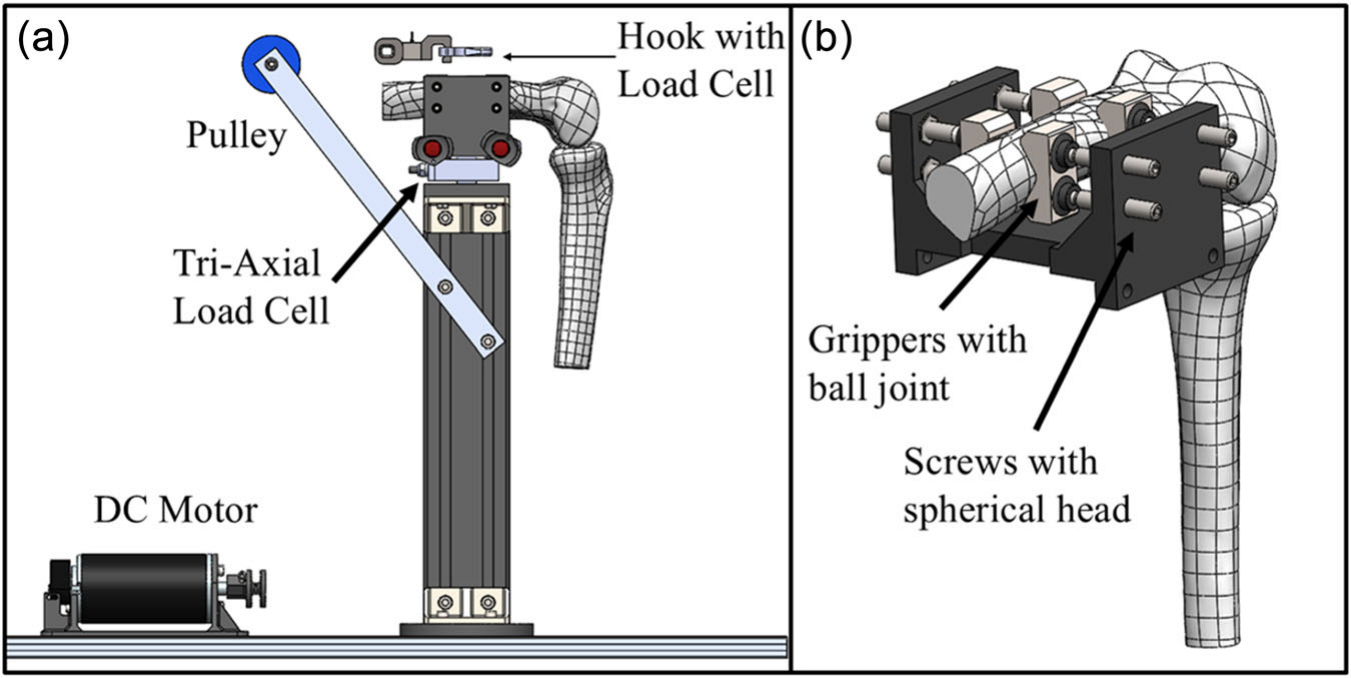
Mechanical module: (a) side view and (b) femur locking frame. DC, direct current.

**Figure 4:**
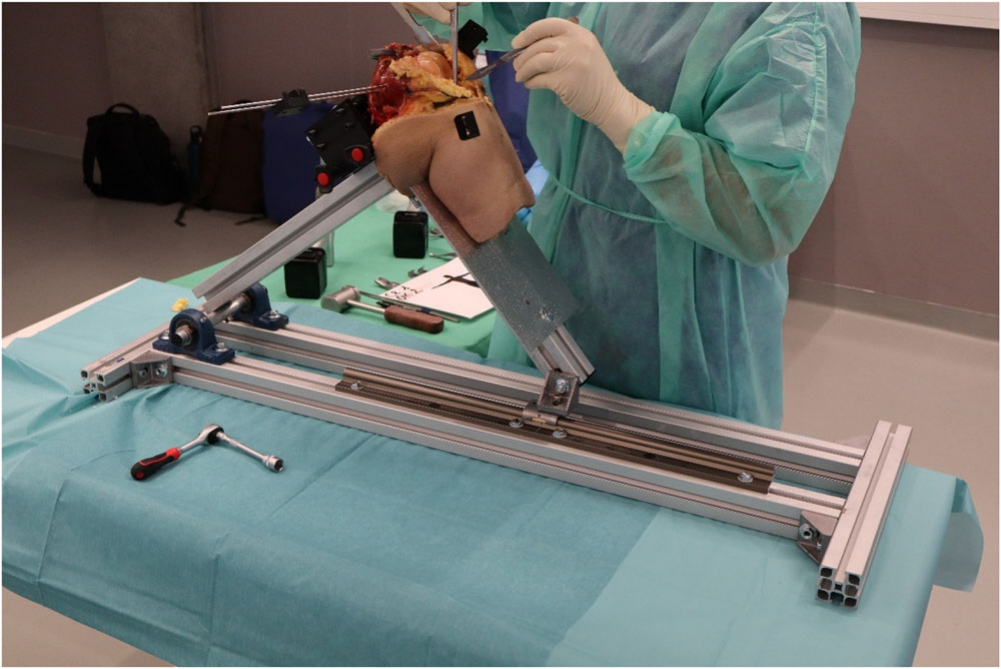
Surgical support metal frame.

The mechanical module (Figure 3) was designed to ensure modularity and portability. The base plates and the testing column can be fully detached from the support metallic table, and can be mounted on a standard medical laboratory table using clamping fixtures, allowing experimental testing in non‐engineering‐specific environments (Figure 3a). The main support column is adjustable and permits testing of both right and left knee specimens. After initial full‐leg landmark registration, the specimen is prepared by amputating the femoral and tibial bones approximately 20 cm from the joint centre, allowing proper insertion and secure fixation within the fixation system. Specimen fixation is achieved through a rigid anchoring system consisting of spherical‐headed clamping arms engaging with toothed fixation plates, providing stable bone fixation and preventing slippage during testing (Figure 3b). The orientation of the femur can be adjusted according to specimen morphology to ensure proper alignment with the quadriceps direction. The fixation block incorporates a quick‐release mechanism with fast locking pins, enabling rapid detachment of the specimen from the testing column and transfer to a surgical frame for intraoperative procedures. An overhanging pulley system mounted on the support column is used to transmit motor‐generated quadriceps loading from the motor to the quadriceps tendon (Figure 3a). The pulley position is adjustable in height to accommodate different knee morphologies and specimen sizes. The mechanical module also includes a dedicated surgical support metal frame (Figure 4), designed to allow surgical procedures to be performed under geometrical and positional conditions comparable to those of the operating environment. The inclination of the frame, resembling the flexion of the lower limb, is adjustable, enabling smooth and precise control of the knee flexion angle according to the specific surgical phase being simulated.

### Actuation and force measurement module

The actuation and force measurement module involves the knee extension system, comprising a DC motor (Maxon DC motor 353297; Maxon Motor AG) connected to the quadriceps through a rectangular loop around which the quadriceps tendon is sutured. The loop is then connected to the motor via a cable and a hook. The motor is used to apply flexion–extension (FE) motion to the joint. All electronic components are contained in a protective hard‐shell transport case for portability. Through a dedicated touchscreen graphical interface, the operator can calibrate the system, configure test parameters and execute predefined or custom testing protocols, including quasi‐static and dynamic knee tests. The interface allows the definition of two active motion protocols: a continuous sequence of FE cycles and a double dynamic flexion (DDF) test, reproducing the knee flexion pattern observed during the gait cycle. In addition, a recorded passive flexion test can be performed manually by the surgeon to replicate the intra‐operative assessment of knee stability and tracking. Force measurement is performed using two load cells. A tri‐axial load cell (F3G‐500N; Forsentek Co.), integrated into the frame, is used to record femoral reaction forces during testing, in particular when performing collateral ligament laxity test. In addition, a mono‐axial load cell (ATO‐LC‐S04; ATO) is embedded in the quadriceps tendon loading path to measure the applied quadriceps force required to generate the prescribed motion. All electronic components for force measurement are housed within the same protective transport case, ensuring system compactness and portability. All measurement data are recorded for subsequent analysis and used to evaluate the forces acting on the knee across different testing configurations.

### Software module

**Figure 5:**
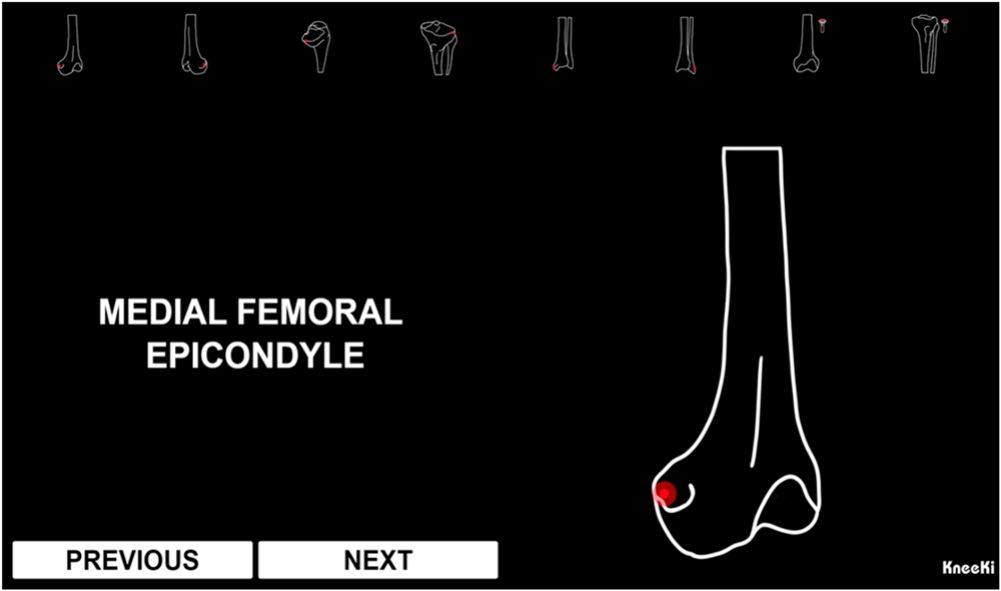
Graphical interface of anatomical landmark registration procedure.

The Software module consists of custom‐developed software specifically designed for the system and entirely implemented in the MATLAB environment (MathWorks Inc.). The software provides full control of the experimental workflow, from anatomical landmarks registration to data processing, result visualization and analysis. The interface was designed to place the system directly under surgeon control, minimizing the need for engineering involvement and enabling fully autonomous operation. This design allows the execution of experimental tests under conditions that closely replicate routine intra‐operative workflows, thereby preserving natural surgical techniques consistent with those applied during real clinical procedures. In addition, dedicated algorithms enable real‐time visualization of tibio‐femoral and patella‐femoral kinematics through the reconstruction of knee geometry based on anatomical landmarks. The anatomical landmark registration procedure (Figure 5), which is similar to those of robotic or navigation systems for knee surgeries, can be performed autonomously by the expert surgeon following the software‐guided protocol and using the digitizing probe in combination with a foot pedal to acquire the landmark points. A total of five points is acquired for the femur, six points for the tibia and four points for the patella. The biomechanical reconstruction of the knee is based on the Grood and Suntay joint coordinate system [13], with corrections proposed by Dabirrahmani and Hogg [5], which is the gold standard for knee kinematic analysis. For the patella, the anatomical reference system proposed by Innocenti et al. [15] was adopted.

**Figure 6:**
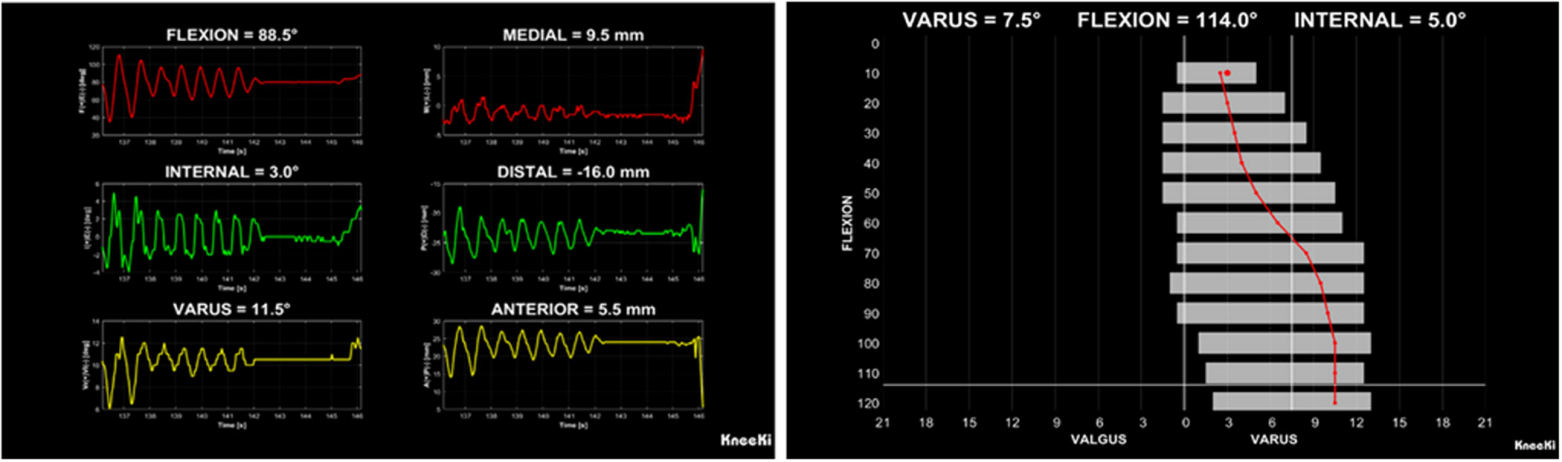
Left: Graphical interface of real‐time data from dynamic test. Right: Graphical interface of real‐time data from laxity test.

A control panel provides full management of the testing process, as well as a preview of the results of the just‐recorded test. The system supports automatic recording of two types of tests: a dynamic test and a laxity test (Figure 6). During the dynamic test, knee motion can be reproduced either passively or actively, as previously described. The software simultaneously analyses and records tibio‐femoral and patello‐femoral kinematics in real time, enabling immediate visualization of the results both during and at the end of the test. All data are automatically stored and readily available for immediate or further analysis. On the other hand, the laxity test is designed to quantify the mechanical response of the knee collateral ligaments. This assessment provides essential information for evaluating ligament balance and optimizing prosthesis alignment during implantation. The software incorporates a predefined protocol that guides the user through both passive and active laxity evaluations, ensuring accurate and reproducible data acquisition.

**Figure 7:**
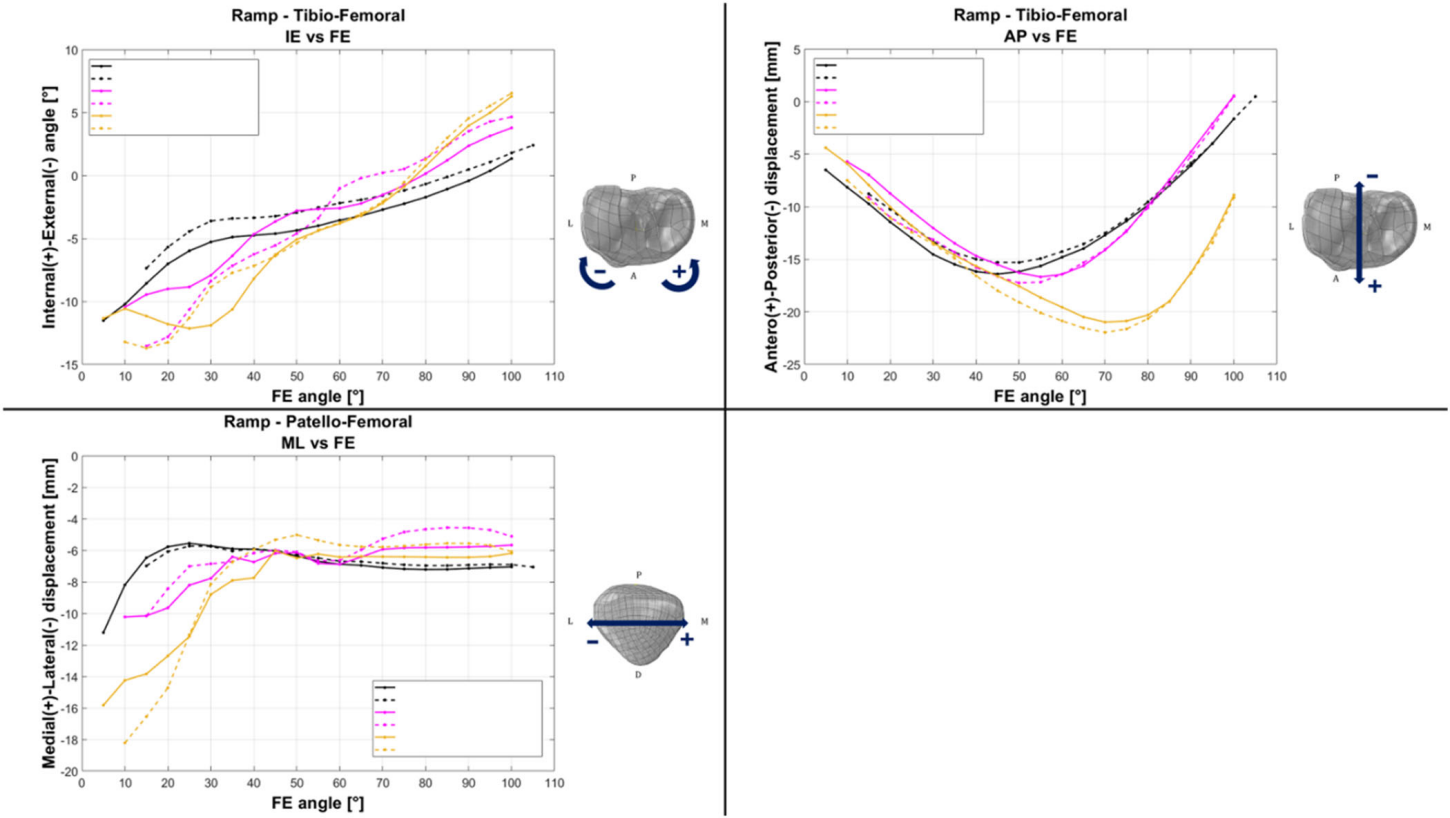
Example of result presentation using the Software module, illustrating the comparison among different test configurations performed on the same specimen. Different colours correspond to different configurations; however, for confidentiality reasons, configuration labels have been anonymized in the legend. Solid lines represent the flexion phase, whereas dashed lines indicate the subsequent extension phase. AP, antero‐posterior; FE, flexion–extension; IE, internal–external; ML, medio‐lateral.

All rotations and translations of both the tibio‐femoral and patello‐femoral joints can be visualized and analysed. For data inspection and analysis, visualization is primarily focused on the most clinically relevant parameters, including tibio‐femoral FE, internal–external (IE) rotation, varus–valgus (VV) rotation and antero‐posterior (AP) translation, as well as patello‐femoral FE and medio‐lateral (ML) translation (Figure 7).

### Validation of system accuracy, repeatability and performance

Validation process was divided into two stages: in vitro and ex vivo testing. During in‐vitro testing, a synthetic knee specimen with rotating hinged prosthesis was used to assess force measurement and motor actuation. The use of a synthetic knee was intended to identify system‐dependent variability, eliminating specimen‐related effects that may emerge from cadaveric tissues' biomechanical changes over time due to degradation. The rotating hinged prosthesis was selected to minimize tibio‐femoral relative displacements during force application, thereby enabling direct loading transfer to the load cell. Motion capture accuracy was ensured by the camera system and verified after each calibration procedure to comply with the predefined maximum error threshold of 0.2 mm. Subsequently, ex vivo testing sessions on cadaveric specimens, representing the actual target of investigation for this system, were conducted to evaluate transportability, setup time, potential reductions in motion capture accuracy under varying lighting conditions, overall performance, workflow smoothness and experimental repeatability.

### In vitro and ex vivo validation

Force measurement accuracy validation was conducted by applying controlled forces under multiple loading configurations on a synthetic knee model with a hand‐held dynamometer (Handifor 20; Tractel). Five repetitions for each configuration were performed. For the mono‐axial load cell, a 50 N tensile force was applied through the cable connecting the motor and the load cell. For the tri‐axial load cell, ML and AP forces of 40 N were applied to the tibia at 0° and 90° of knee flexion. The validation protocol on the tri‐axial load cell was designed to replicate clinically relevant manual laxity tests. Force direction was controlled using a goniometer with an angular accuracy of 0.5°, ensuring consistent and repeatable load application across test conditions. Motor actuation was evaluated by applying FE cycles and DDF tests on the synthetic knee model. The FE test included 5 s of flexion phase, 5 s of intermediate resting phase and 5 s of subsequent extension phase, for a total duration of 15 s, spanning a range of motion from 0° to 90°. This protocol replicates the FE test used during ex vivo experimental measurements. DDF test was conducted, consisting of three repetitions with a duration of 1.5 s for each phase, ranging from 5° to 16° and then to 58°. The motor operates linearly between predefined angular limits and was calibrated using a goniometer with an accuracy of 0.5°, double‐checking the data with the motion capture kinematic outputs. Five repetitions were recorded for each test, focusing primarily on the FE angle as a reference variable to calculate mean and standard deviation induced by the error of the motor. Subsequently, the ex vivo validation was conducted, comprising five testing sessions and a total of nine cadaveric specimens. Setup time was recorded in all sessions, motion capture accuracy was verified in each environment, and repeatability was quantified based on output data. Any critical failure leading to a significant slowdown of the tests or preventing further progression was systematically documented. In each session, at least one senior surgeon was present and directly operated the system to assess its usability.

## RESULTS

The results of the force measurement validation protocol are summarized in Table 1, which reports the set and measured force values with 95% confidence intervals for each loading configuration, including both positive and negative directions. Across all loading configurations, the measured forces were close to the set values, with narrow 95% confidence intervals. The quadriceps tendon load cell showed minimal deviation from the set load. Medio‐lateral and AP force measurements demonstrated consistent performance at both 0° and 90° of knee flexion, with slightly higher variability observed during ML loading.

**Table 1:** Set and measured value with 95% confidence interval for each configuration both in positive and negative directions.

Configuration	Set	Measured (+)	Measured (−)
Quadriceps tendon load cell	50 N	50.1 N ± 0.5 N	N/A
ML stress at 0° FE	40 N	42.8 N ± 3.6 N	40.6 N ± 2.7 N
ML stress at 90° FE	40 N	39.7 N ± 0.6 N	43.7 N ± 1.5 N
AP stress at 0° FE	40 N	37.0 N ± 3.1 N	37.1 N ± 1.2 N
AP stress at 90° FE	40 N	40.7 N ± 3.2 N	39.9 N ± 1.2 N

*Note*: Considering the load cell system of reference, ML forces are positive along the lateral direction for a left knee, while for a right knee, they are positive along the medial direction. Regarding AP forces at 90°, both for left and right knee, the forces are considered positive if they are applied in the posterior direction, while at 0°, they are positive if they are applied in the anterior direction.

Abbreviations: AP, antero‐posterior; FE, flexion–extension; ML, medio‐lateral; N/A, not applicable.

**Figure 8:**
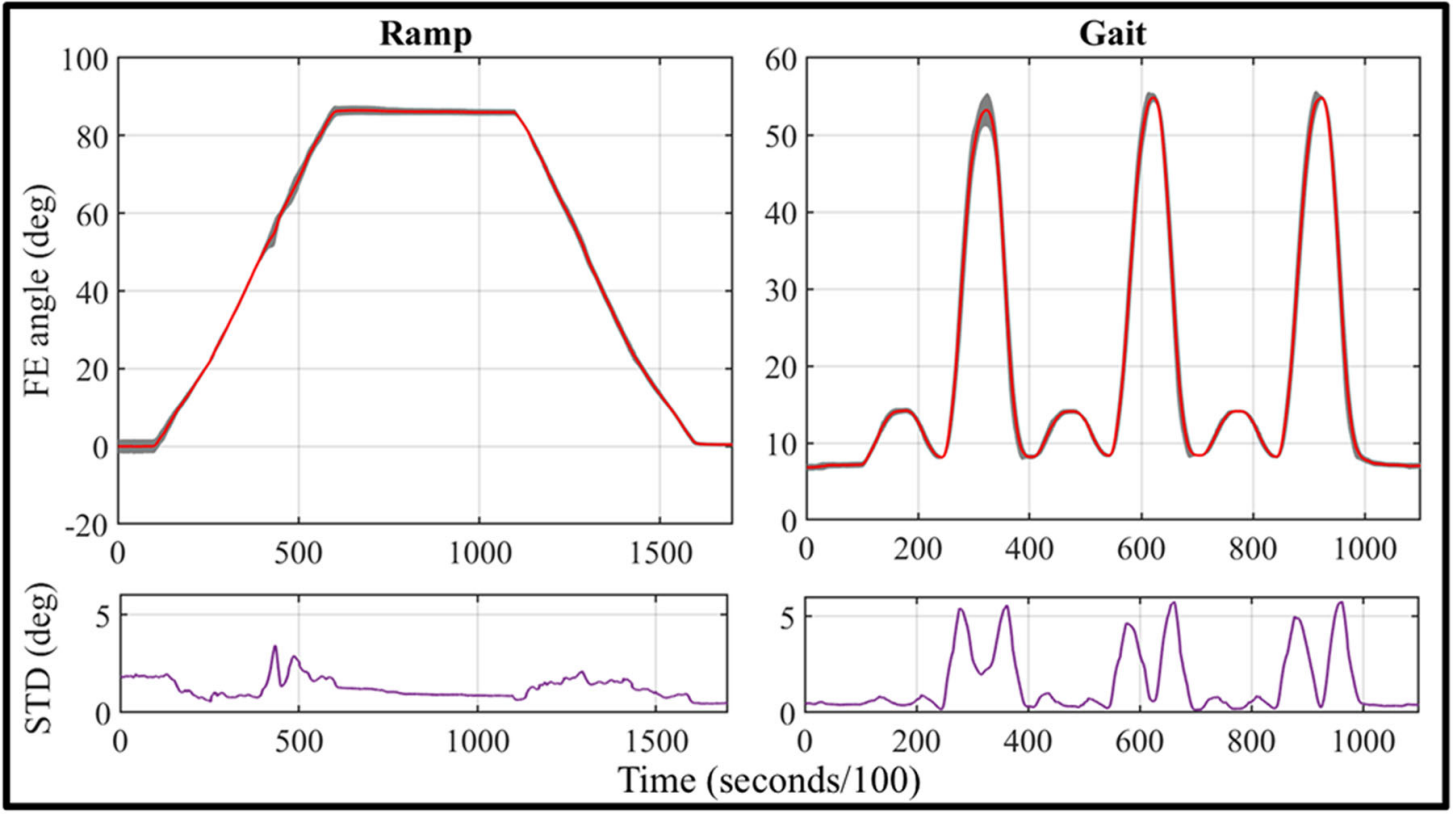
Mean values (red) with STD area (grey) and actual STD value (purple) for both FE and DDF motion. DDF, double dynamic flexion; FE, flexion–extension; STD, standard deviation.

Motor actuation exhibited high repeatability, with better performance observed in FE cycles compared to DDF tests (Figure 8). In the former, the actual standard deviation did not exceed 5°, with a mean value of 0.51°. In the latter, characterized by faster and more dynamic motion on the single sub‐cycle, the actual standard deviation exceeded 5° at peak velocity phases, while still maintaining a mean value of 1.62°. The accuracy was slightly affected by the dynamic behaviour of the DDF, which reduced the requested peak values of 16° and 58° resulting in an overall smaller range.

**Figure 9:**
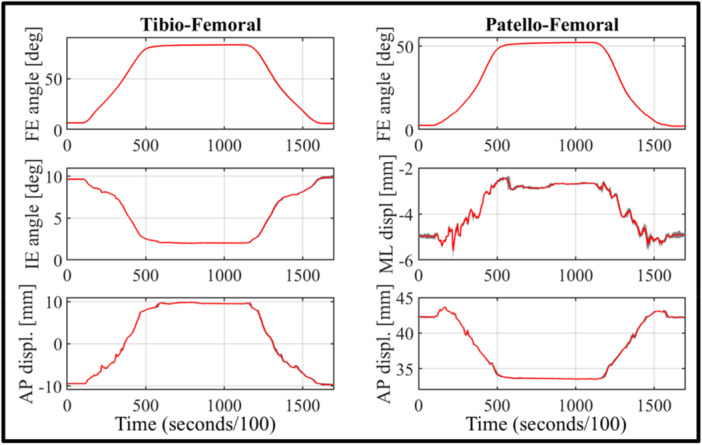
Flexion–extension motion: relevant measurement for tibio‐femoral and patello‐femoral joints; red line is the average value, grey area is the standard deviation. AP, antero‐posterior; FE, flexion–extension; IE, internal–external; ML, medio‐lateral.

**Figure 10:**
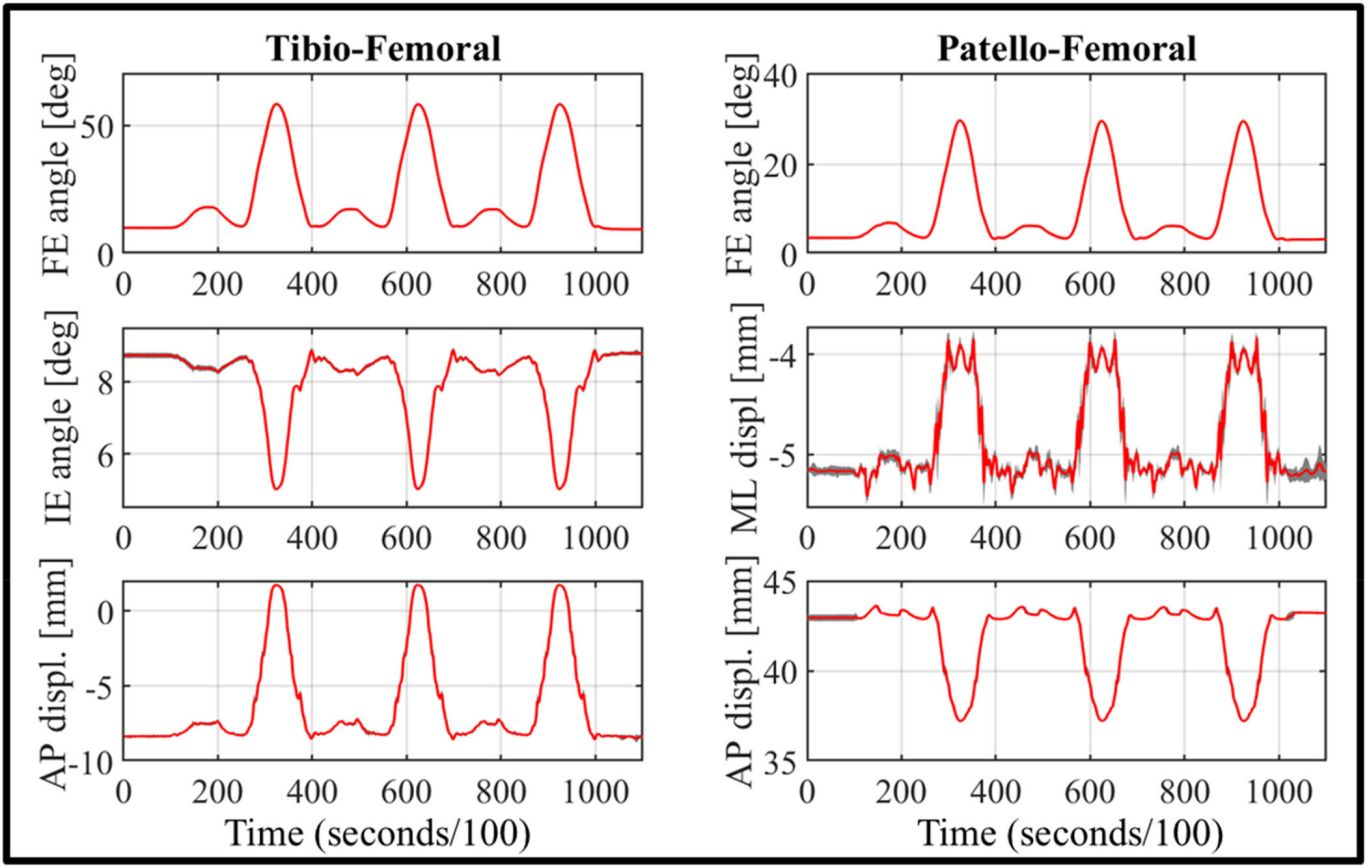
DDF motion: relevant measurement for tibio‐femoral and patello‐femoral joints; red line is the average value, grey area is the standard deviation. AP, antero‐posterior; DDF, double dynamic flexion; FE, flexion–extension; IE, internal–external; ML, medio‐lateral.

For the ex vivo testing sessions, the system was easily transported either using a medium‐sized car or shipped via standard delivery services. For all five sessions, the complete setup always required less than 1 h with one person working on it. With the current configuration, motion capture system calibration always achieved a pointer tip positional error of a maximum of 0.200 mm (range: 0.147–0.198 mm), regardless of the light environment. A decrease in accuracy was registered in every testing session after a continuous time of utilization of approximately 4 h, due to the overheating of the cameras and overload of the system. In all cases, the issue was successfully resolved by leaving the system powered off for a few minutes, rebooting and subsequently repeating the calibration procedure to restore the initial accuracy. The overall process took, in each case, 21 min on average (range: 19–23 min). Throughout all the testing sessions, no critical failure was registered, and each session was completed as expected. Figures 9 and 10 show the overall performance results for the relevant tibio‐femoral and patella‐femoral degrees of freedom, extracted from a representative specimen: the red line represents the average value and the grey shade the standard deviation. Flexio‐extension motion was limited between 6.1° and 84.8° due to the specimen stiffness, compared to the requested range of 0°–90°. DDF dynamic was more accurately replicated with peak points of 16.6° and 57.8°, thanks to the narrower range that was not affected by specimen stiffness. The baseline value was the most different one, at 9.37°. Nevertheless, the mean standard deviation values of all rotational and translational results obtained from FE and DDF tests in the cadaveric study are reported in Table 2.

**Table 2:** Average standard deviation for all rotations and translations, for both the tibio‐femoral and patello‐femoral joint.

	FE	AA	IE	ML	AP	PD
FE Tibio‐Femoral	0.43°	0.02°	0.07°	0.07 mm	0.14 mm	0.08 mm
FE Patello‐Femoral	0.22°	0.06°	0.16°	0.17 mm	0.09 mm	0.19 mm
DDF Tibio‐Femoral	0.45°	0.04°	0.07°	0.08 mm	0.17 mm	0.09 mm
DDF Patello‐Femoral	0.29°	0.13°	0.14°	0.15 mm	0.12 mm	0.18 mm

Abbreviations: AA, abduction–adduction; AP, antero‐posterior; DDF, double dynamic flexion; FE, flexion–extension; IE, internal–external; ML, medio‐lateral; PD, proximal–distal.

## DISCUSSION

This study presents the design and aims to validate a portable, surgeon‐oriented experimental system for the biomechanical assessment of knee joint kinematics and laxity. The validation results indicate that the proposed platform provides reliable and repeatable measurements of both kinematic and kinetic variables under controlled experimental conditions. Force measurements obtained from the load cell validation protocol showed close agreement with the applied loads across all configurations, with limited variability and narrow confidence intervals. The mono‐axial load cell achieved a measurement accuracy of approximately 1% of full scale (FS = 200 N). Given that quadriceps tendon forces measured during testing reach values of up to 130 N, this level of accuracy is sufficient for analysing the relationship between knee flexion–extension angle and quadriceps tendon force, and it is consistent with the performance reported for experimental systems employing similar quadriceps tendon actuation strategies [20]. This capability supports comparative analysis of quadriceps force requirements before and after surgical intervention, such as prosthetic implantation. The tri‐axial load cell achieved a measurement accuracy of approximately 0.75% of full scale (FS = 500 N) when measuring externally applied knee forces. The slightly higher dispersion observed during ML loading is consistent with the manual nature of clinically relevant laxity tests and does not compromise measurement reliability. This level of accuracy allows reliable laxity assessment through combined analysis of applied loads and corresponding kinematic responses, and it is consistent with the performance reported for comparable experimental devices [8]. Motor‐driven motion repeatability was confirmed through both flexion–extension and DDF tests, with low standard deviation values across repeated trials. The comparison between motor‐estimated and motion‐capture‐measured flexion angles demonstrated small static discrepancies and limited dynamic variability, supporting the adequacy of the actuation system for reproducible kinematic testing. Across five motion repetitions, the average standard deviation was 0.51° for the ramp motion and 1.62° for the DDF protocol. These values are comparable to, or lower than, those reported for more complex and less versatile experimental systems [11, 21, 23]. It should be noted that, while the actuation system does not provide absolute positional accuracy, the kinematic results are always referenced to the motion capture measurements, which represent the ground truth for joint position [16]. Accordingly, the reported kinematic accuracy reflects the measurement capability of the system rather than the intrinsic precision of the motor, which primarily serves to reproducibly drive the joint motion. Overall, these findings demonstrate that the system achieves a level of accuracy and repeatability suitable for experimental investigation of knee joint biomechanics.

Ex vivo testing demonstrated that the system is highly portable, with complete setup achieved within a short time, thereby maximizing the time available for the execution of the actual tests. The compact and modular design enabled successful operation in cadaveric laboratories that may not be perfectly suited for biomechanical studies, while maintaining spatial accuracy below 0.20 mm, which is comparable to values previously reported in the literature [2]. This level of portability extends the applicability of biomechanical testing beyond conventional biomechanics laboratories, facilitating experimental investigations in settings that more closely reflect the surgeon's working environment. Such flexibility is particularly relevant for collaborative research involving clinical centres and implant manufacturers, as well as for experimental setups where space, infrastructure or the presence of fixed systems would limit the use of larger and more complex platforms. The observed decrease in accuracy following prolonged continuous use was expected for this type of system. The required interruption is fully compatible with standard testing workflows, as it can be accommodated within typical half‐day breaks. Moreover, the recovery time averages approximately 20 min, representing a limited downtime that does not compromise the overall session efficiency. The actuation system provided sufficient power and robustness to reliably drive knee motion in cadaveric specimens, with peak quadriceps tendon forces exceeding 130 N during testing. The kinematic data obtained from the ex vivo study showed high trajectory repeatability, as reflected by the low mean standard deviation of the outputs across five repetitions of both flexion–extension and DDF motions. These results indicate consistent and reliable reproduction of knee motion under realistic experimental conditions, even in the presence of intrinsic resistance commonly observed in cadaveric specimens, which the system was able to effectively overcome.

A further strength of the proposed system is its ability to accurately capture patella‐femoral kinematics in addition to tibio‐femoral kinematics. This capability enhances the biomechanical relevance of the experimental assessment, as patella‐femoral biomechanics plays a critical role and remains usually relatively under‐investigated compared with tibio‐femoral kinematics. In addition, the platform enables systematic analysis of the native knee condition for each specimen prior to surgical intervention, providing a specimen‐specific reference configuration that serves as a control for subsequent comparisons. This patient‐specific baseline allows the biomechanical effects of surgical procedures or implant configurations to be evaluated relative to the native state of the same specimen. Overall, the ex vivo validation confirms that the platform provides the accuracy, repeatability and practical applicability required for advanced biomechanical studies of the knee joint.

Some limitations of the present study should be acknowledged. At the current stage, the platform does not support closed‐chain motion testing, and the experimental evaluation was therefore limited to open‐chain conditions. Quadriceps activation was implemented through a single cable‐driven actuation, which does not reproduce the physiological synergy among the different quadriceps heads. In addition, specimen preparation required bone amputation, resulting in the loss of surrounding muscular soft tissues. However, all experimental conditions were systematically compared with the native configuration of the same specimen, such that any potential effect related to specimen preparation was consistently present across configurations and therefore did not bias the comparative analysis. Lastly, variability in landmark registration was not assessed, as it is primarily dependent on surgeon experience and skill, as well as specimen‐specific deformities, rather than the system itself. Future studies will investigate the impact of this variability on the computed kinematics.

## CONCLUSION

This study introduced and validated a portable and surgeon‐oriented experimental platform for the biomechanical assessment of knee joint kinematics and laxity under intraoperative‐like conditions. The system demonstrated reliable and repeatable measurement of both kinematic and kinetic variables, together with sufficient accuracy for comparative biomechanical investigations in ex vivo settings. The modular design, rapid setup and surgeon‐oriented workflow allow experimental testing to be conducted in environments that closely resemble clinical practice, overcoming key limitations of traditional, fixed biomechanics laboratories. By enabling combined analysis of tibio‐femoral and patella‐femoral kinematics, force measurements and specimen‐specific native reference configurations, the platform provides a comprehensive and clinically meaningful framework for evaluating the biomechanical impact of surgical decisions and implant configurations. Overall, this system represents a versatile and practical tool for advanced knee biomechanics research, with potential applications in implant development, surgical technique optimization and intraoperative strategy assessment.

## AUTHOR CONTRIBUTIONS

*Conceptualization*: Mattia Sisella and Bernardo Innocenti. *Methodology*: Mattia Sisella, Lorenzo Maggi and Bernardo Innocenti. *Formal analysis and investigation*: Mattia Sisella and Lorenzo Maggi. *Writing—original draft preparation*: Mattia Sisella, Lorenzo Maggi. *Writing—review and editing*: Mattia Sisella, Lorenzo Maggi, Benedetto Allotta and Bernardo Innocenti. *Funding acquisition*: Benedetto Allotta, Bernardo Innocenti. *Resources*: Bernardo Innocenti. *Supervision*: Bernardo Innocenti.

## CONFLICT OF INTEREST STATEMENT

The authors declare no conflict of interest.

## ETHICS STATEMENT

No identifying information related to donors was available to the investigators.

## Data Availability

Additional data can be provided upon request.
